# Tracking of Normal and Malignant Progenitor Cell Cycle Transit in a Defined Niche

**DOI:** 10.1038/srep23885

**Published:** 2016-04-04

**Authors:** Gabriel Pineda, Kathleen M. Lennon, Nathaniel P. Delos Santos, Florence Lambert-Fliszar, Gennarina L. Riso, Elisa Lazzari, Marco A. Marra, Sheldon Morris, Asako Sakaue-Sawano, Atsushi Miyawaki, Catriona H. M. Jamieson

**Affiliations:** 1 Divisions of Regenerative Medicine and Hematology-Oncology, Department of Medicine, Moores Cancer Center, University of California, San Diego, La Jolla, CA 92093-0820, USA; 2 Biological Sciences Department, California Polytechnic State University, San Luis Obispo, CA, 93407, USA; 3 Doctoral School of Molecular and Translational Medicine, Department of Health Sciences, University of Milan, Milan, Italy; 4 Canada’s Michael Smith Genome Sciences Centre, BC Cancer Agency, Vancouver, BC, Canada; 5 Laboratory for Cell Function and Dynamics, Brain Science Institute, RIKEN, Wako-city, Saitama, Japan

## Abstract

While implicated in therapeutic resistance, malignant progenitor cell cycle kinetics have been difficult to quantify in real-time. We developed an efficient lentiviral bicistronic fluorescent, ubiquitination-based cell cycle indicator reporter (*Fucci2BL*) to image live single progenitors on a defined niche coupled with cell cycle gene expression analysis. We have identified key differences in cell cycle regulatory gene expression and transit times between normal and chronic myeloid leukemia progenitors that may inform cancer stem cell eradication strategies.

Malignant reprogramming of progenitors into self-renewing cancer stem cells (CSCs), which have a proclivity for dormancy in protective niches, has been implicated in therapeutic resistance of chronic myeloid leukemia (CML) and other CSC-driven malignancies^1^,^2^,^3^,^4^,^5^. A pressing unmet medical need for developing therapies that target niche dependent dormant human CSCs provides a compelling rationale for identifying key differences in gene expression at different cell cycle phases between live normal and malignant progenitors in a CSC-supportive stromal co-culture system^2^,^6^,^7^,^8^,^9^,^10^.

## Results

### Normal and Malignant Progenitor Cell Cycle Transcriptomic Analysis

**Figure 1:**
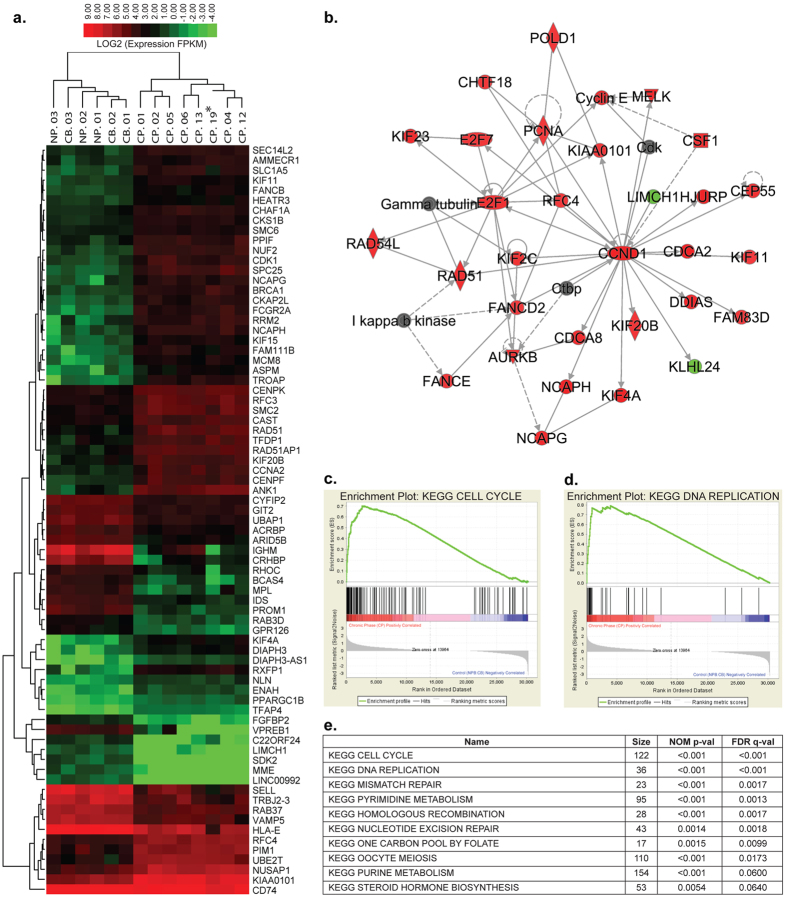
RNA-seq identifies distinct differences in DNA replication and cell cycle gene expression in normal and chronic phase CML progenitor cells. (**a**) Heatmap from agglomerative hierarchical clustering of GSEA top ranked genes between 8 chronic phase CML (CP), 3 normal peripheral (NP) blood and 3 cord blood (CB) samples using RNA-seq data. Red indicates higher expression values and green indicates lower expression values (Log2 FPKM scale). (**b**) Ingenuity^®^ Pathway Analysis performed on statistically significant genes (p < 0.05) between CP (n = 8) and normal (n = 6, 3 NP, 3 CB): RNA-seq expression data reveals cyclin D1 (CCND1) as a major hub. Arrows indicate interaction directionality. Red indicates increased expression and green indicates decreased expression in CP relative to NP. (**c,d**) Enrichment plots for KEGG Cell Cycle and DNA Replication pathways from Gene Set Enrichment Analysis (GSEA) between 8 CP and 6 normal (3 CB, 3 NP) samples using RNA-seq gene expression data indicate enrichment of these pathways in CP relative to NP. (**e**) GSEA Summary table obtained from RNA sequencing data comparing 8 CP to 6 normal (3 CB, 3 NP) samples confirms Cell Cycle and DNA Replication pathways as significant to CP and normal differential expression.

To provide insights into differences between normal and chronic phase (CP) CML progenitor cell cycle regulation, cell cycle regulatory gene expression changes were quantified using whole transcriptome RNA sequencing (RNA-seq) of fluorescence-activated cell sorting (FACS)-purified CP CML, normal peripheral blood (NP), and cord blood CD34^+^ CD38^+^ Lin^**−**^ progenitors. Agglomerative hierarchical clustering analysis (Fig. 1a), and Ingenuity^®^ Pathway Analysis (IPA, QIAGEN Redwood City, www.qiagen.com/ingenuity) revealed key hubs, including CCND1, PCNA, E2F1, RAD51, FANCD2, and AURKB, representing cell cycle, cancer, and cellular movement associated genes that distinguished CP CML from normal progenitors (Fig. 1b). Of all pathways considered in RNA-seq analysis, Gene Set Enrichment Analysis (GSEA) revealed that the Cell Cycle and DNA Replication KEGG pathways (Nominal p-value < 0.001) were most enriched in CP CML compared with normal progenitors (Fig. 1c–e). An IPA was also performed for genes represented in a PCR cell cycle array from each RNA-seq experiment (CP and normal) (Supplementary Fig. 1a). In keeping with RNA-seq data, major hubs in this network represented cell cycle check points and DNA damage response genes (CHEK1, CHEK2, Cyclin B, RAD51, MRE11A, ATR, and ATM)^11^.

### Lentiviral Bicistronic Fucci2BL Quantification of Cell Cycle Kinetics

Currently, few methods exist for quantifying cell cycle progression and analyzing regulators of gene expression in live human normal and malignant progenitors. To date, efficient cell cycle transit time analysis in single live human progenitors derived from primary patient samples has been hampered by (1) decreased cell viability following transfection or transduction, (2) limited sample size, (3) dormancy of primitive progenitor populations thereby necessitating lentiviral rather than retroviral transduction and 4) increased apoptosis in the absence of a supportive microenvironment. Current methods require genetic manipulation, sequential transduction, and clonal selection^12^,^13^,^14^,^15^,^16^. However, these methods and conditions are relatively inefficient for comparative studies involving primary patient sample-derived CSCs and their normal progenitor counterparts.

**Figure 2:**
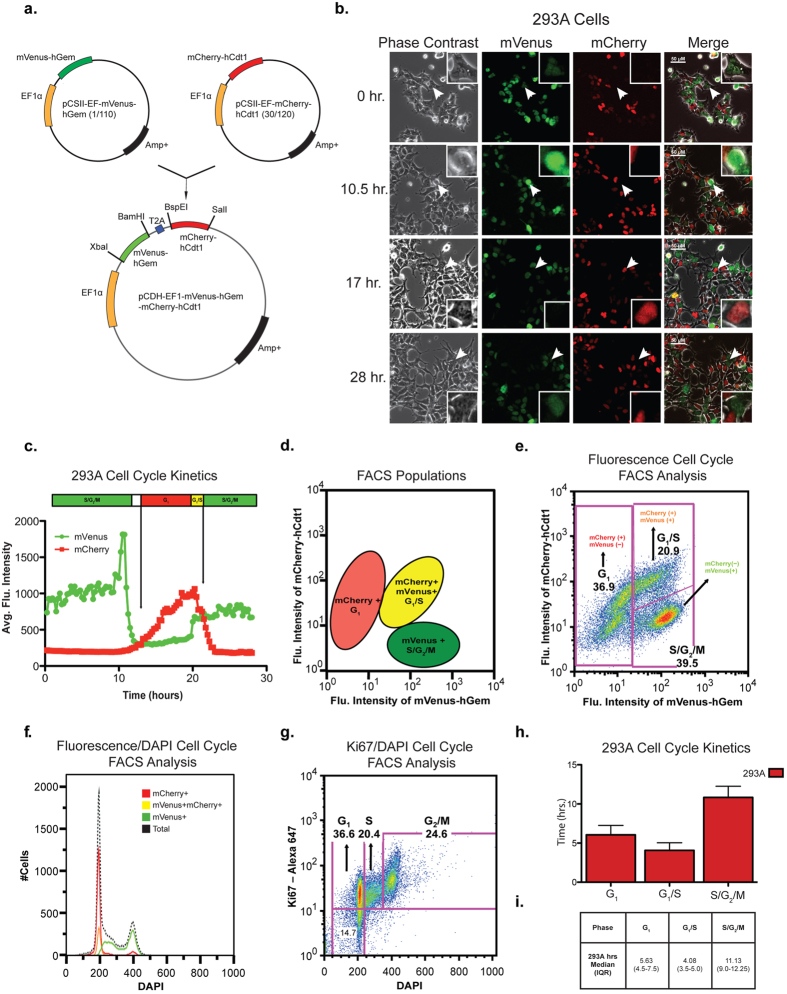
Fucci2BL vector generation and characterization. (**a**) Diagram and map of construct design and generation. Both mVenus-hGem(1/110) and mCherry-hCdt1 (30/120) were subcloned into a pCDH-EF1α-T2A lentiviral expression vector. (**b**) Temporal analysis of confocal images generated from lentiviral transduced 293A cells stably expressing fluorescent reporters. White arrows mark which cell was tracked to analyze cell cycle kinetics. (**c**) Cell cycle kinetics was determined from average fluorescence intensity from marked cells expressing reporters. (**d**) Diagram representing location of specific cell populations (mCherry^+^, mCherry^+^mVenus^+^, mVenus^+^) identified by FACS analysis. (**e**) Live cell FACS analysis of 293A cells stably expressing fluorescent reporters. (**f**) DNA content analysis using DAPI stain on 293A cells stably expressing fluorescent reporters. (**g**) Cell cycle FACS analysis using Ki-67 staining and DAPI on 293A cells stably expressing fluorescent reporters. (**h**) Cell cycle kinetics of 293A (n = 10) cells represented in hours. (**I**) Duration of cell cycle phase represented by median (hours) for 293A (n = 10) cells.

To alleviate this challenge and improve transduction efficiency, we generated a lentiviral bicistronic reporter vector encoding fluorescent ubiquitination-based cell cycle indicator probes (Fucci2). The ***Fucci2BL*** lentiviral vector expresses mVenus-hGem(1/110) fused to mCherry-hCdt1(30/120) by the T2A peptide using an EF1 promoter that generates optimal levels of gene expression in progenitors^17^ (Fig. 2a). These fluorescent reporters can distinguish three cell cycle phases as G_1_ by red fluorescence, G_1_/S by yellow fluorescence, and S/G_2_/M by green fluorescence. Although these reporters cannot specifically distinguish between G_1_ and G_0_ it has been previously demonstrated that red fluorescent signal intensities generated from mCherry-hCdt1(30/120) reporter in particular can enrich for cells in G_1_ and quiescent G_0 _cells^18^. This lentiviral bicistronic vector ensures that ubiquitin regulated cell cycle sensors are expressed equally. Initially, the Fucci2BL vector transduction efficiency and the fidelity of cell cycle kinetic analysis were compared with 293A cells that were co-transduced with both mVenus-hGem(1/110) and mCherry-hCdt1(30/120) independent Fucci2 reporters (Supplementary Fig. 2a). Notably, 293A cells transduced with our Fucci2BL reporter displayed distinct nuclear staining of either green or red fluorescence and normal cell morphology. Transduction efficiency appeared to be higher with the single vector Fucci2BL compared with the standard sequential transduction schema^12^,^19^. Moreover, single transduction with the Fucci2BL bicistronic expression vector would be expected to better preserve primary progenitor viability. Next we characterized the fidelity of cell cycle in 293A cells transduced with the Fucci2BL reporter, which stably express mVenus-hGem(1/110) and mCherry-hCdt1(30/120), using time-lapse confocal fluorescence microscopy. These 293A cells revealed normal cell morphology and distinct nuclear staining of either green or red fluorescence depending on the cell cycle stage with red fluorescence indicating G_1_, yellow indicating G_1_/S and green fluorescence indicating S/G_2_/M (Fig. 2b; Supplementary Fig. 2b). In 293A cells, the duration of each cell cycle phase was determined by quantifying the average fluorescence intensity in individual live cells by confocal fluorescence microscopy following Fucci2BL reporter transduction (Fig. 2c,d). FACS analysis was used to quantify the percentage of cells in each phase of the cell cycle. Based on FACS analysis, 36.9% of cells are in G_1_, 20.9% in G_1_/S, and 39.5% in S/G_2_/M (Fig. 2e). As expected, mVenus^+^ positive cells are in both G_1_ and S phase, containing double the DNA content of mCherry^+^ and mVenus^+^/mCherry^+^ cells as represented by a two-fold increase in DAPI signal (Fig. 2f). Although both mVenus-hGem(1/110) and mCherry-hCdt1 (30/120) sensors have been previously characterized and validated, it was important to determine both were properly regulated while expressed equally from the lentiviral bicistronic vector. Since the Fucci reporters can distinguish G_1_, G_1_/S, and S/G_2_/M cell cycle phases it was important to confirm the accuracy of the new reporter by comparing it to a validated method^2^ used to study cell cycle status based on Ki-67 and DAPI staining for FACS analysis. As a final method for characterizing the fidelity of our Fucci2BL reporter, stably transduced 293A cells were analyzed using Ki67/DAPI cell cycle FACS analysis. Using this approach, 36.6% of cells were found to be in G_1_, 20.4% in S, and 24.6% in G_2_/M (Fig. 2g). A confocal fluorescence microscopic comparison of cell cycle kinetics of normal progenitor CD34^+^ (NP) cells compared to 293A cells revealed a trend toward prolongation of S/G_2_/M (Supplementary Fig. 2e). The median duration of G_1_ was 5.63 hours (IQR 4.5–7.5), G_1_/S phase was 4.08 hours (IQR 3.5–5.0), and S/G_2_/M was 11.13 hours (IQR 9.0–12.25), for 293A cells (Fig. 2h,i and Supplementary Video 1). Together, these studies demonstrated the high fidelity of the Fucci2BL system with regard to quantification of cell cycle kinetics in cell lines.

### Molecular Characterization of Normal and Malignant Progenitor Cell Cycle Kinetics on a Defined Niche

**Figure 3:**
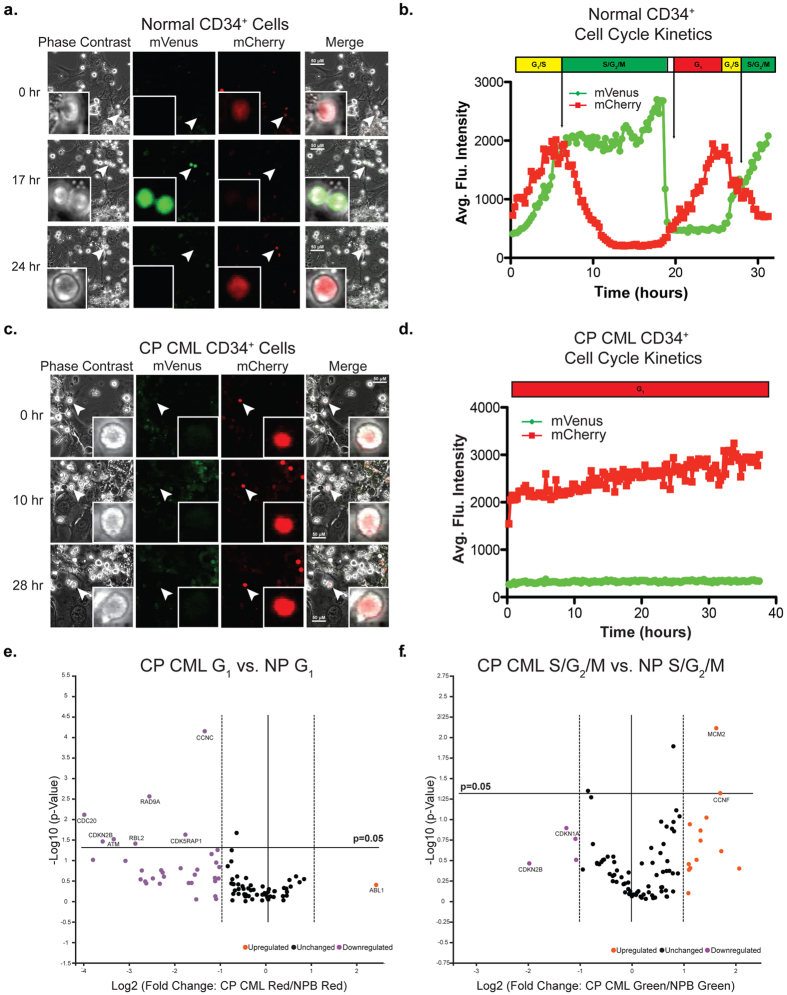
Live cell imaging of stromal co-cultures distinguishes differences in cell cycle kinetics between chronic phase CML (CP) and normal peripheral blood (NP) CD34^+^ cells. (**a**) Temporal analysis of confocal images generated from lentiviral transduced CD34^+^ selected NP cells grown on SL/M2 co-culture with bicistronic fluorescent reporter. White arrows mark which cell was tracked to analyze cell cycle kinetics. (**b**) Cell cycle kinetics was determined from average intensity of fluorescence from marked cells (white arrow) expressing Fucci2BL reporter. (**c**) Temporal analysis of confocal images generated from lentiviral transduced CD34^+^ selected (CP) cells grown on SL/M2 co-culture with bicistronic fluorescent reporter. White arrows mark which cell was tracked to analyze cell cycle kinetics. (**d**) Cell cycle kinetics was determined from average intensity of fluorescence from marked cells expressing reporters. (**e**) Volcano plot of CP CML (G_1_) phase (n = 3) differential cell cycle gene expression compared to NP (G_1_) phase (n = 3). Orange circles indicate genes expressed greater than +1.0 Log2 fold change in CP CML (G_1_) vs NP (G_1_). Magenta circles indicate genes downregulated and expressed less than −1.0 Log2 fold change in CP CML (G_1_) vs NP (G_1_). (**f**) Volcano plot of CP CML (S/G_2_/M) phase (n = 3) differential cell cycle gene expression compared to NP (S/G_2_/M) (n = 3). Orange and magenta indicate same nomenclature as above.

Next, we addressed (1) whether clonal cell cycle kinetic differences could be resolved in live normal versus chronic phase progenitors, (2) whether specific gene expression changes during different phases of the cell cycle could be quantified, and (3) whether cell cycle kinetics differed between normal and CP CML progenitors in a niche-responsive manner. To this end, we transduced CD34^+^ selected progenitors from both human NP and CP CML with the Fucci2BL reporter, followed by culturing on a SL/M2-LSC stromal co-culture system (Supplementary Fig. 3a). On SL/M2 stroma, transduced normal CD34^+^ cells on average transited the cell cycle within 26 hours (Fig. 3a,b and Supplementary Video 2). In contrast, CP CML CD34^+^ cells transduced with Fucci2BL demonstrated a prolongation of transit through G_1_ (Fig. 3c,d). A minor portion was also observed to cycle completely through all phases of the cell cycle. Confocal fluorescent images were obtained at discrete intervals (0, 10hrs, 28hrs), and cell cycle kinetics of CD34^+^ CP CML cells was determined using average fluorescence intensity (Fig. 3c,d and Supplementary Video 3). Quantitative confocal fluorescence microscopic analysis of single normal (NP; n = 19) and CP CML (CP; n = 17) CD34^+^ progenitors was used to generate G_1_ phase averages (Supplementary Fig. 3c). Comparative analysis of cell cycle transit in NP versus CP CML CD34^+^ cells demonstrated that CP CD34^+^ CML cells (11 out of 17 cells) harbored significantly longer G_1_ phase transit times (p-value 0.0014; Fisher’s exact test) compared with normal progenitor counterparts (Supplementary Fig. 3b,c). Three out of the (n = 17) cells were observed to cycle completely through all phases of the cell cycle. Multi-color FACS analysis revealed that the majority of CP CML progenitors remained viable in stromal co-cultures used for confocal fluorescence microscopic imaging of cell cycle status (Supplementary Fig. 3f). Furthermore, Ki-67 and DAPI based-cell cycle FACS analysis confirmed that CP CML CD34^+^ cells transit G_1_-phase slowly compared with their normal progenitor counterparts (Supplementary Fig. 3g). To investigate transcription patterns related to the extended G_1_-phase observed in CP CML progenitors, Integrated System for Motif Activity Response Analysis (ISMARA) was completed on reads from CD34^+^ CP CML (n = 3) and NP (n = 3) samples subjected to RNA-seq and aligned to the GRCh37 reference genome using STAR (https://github.com/alexdobin/STAR). As an online tool, ISMARA identifies key transcription factors responsible for observed genome wide expression patterns based on computationally predicted transcription factor regulatory sites or motifs^20^. The top ranked motifs, designated by Z-value from approximately 200 curated motifs, were those associated with the transcription factors E2F1-5 (Z-value = 5.8) and TFDP1 (Z-value = 4.6), which heterodimerize and regulate the G_1_ to S phase transition. Activity profiles from E2F1-5 and TFDP1 indicated higher activity in CP CML cells compared to NP cells thereby providing a novel insight into the mechanisms governing the extended G_1_-phase observed in CP CML cells (Supplementary Fig. 3d,e).

Next, we utilized the quantitative RT^2^–PCR Profiler Array to monitor the response of 84 genes involved in cell cycle regulation, and compared CD34^+^ CP CML and normal progenitor cells at different phases of the cell cycle (Supplementary Fig. 3a). When comparing FACS-purified CP CML versus normal progenitor (n = 6 samples) from different phases of the cell cycle, PCR array based gene expression differences were observed in G_1_ and S/G_2_/M cell cycle phases. Specifically, examination of the G_1 _cell cycle phase after 72 hours in the stromal co-culture system revealed 8 significantly differentially expressed genes (Fig. 3e). In the G_1_ (red) cell cycle phase, 7 cell cycle regulatory genes, including CDC20, CDKN2B, ATM, RBL2, RAD9A, CDK5RAP1, and CCNC, were significantly (p < 0.05) downregulated in CP CML compared with normal progenitors. Downregulation of CDC20 may lead to deregulation of the APC/C complex coinciding with delayed G_1_/S transition observed in CP CML progenitors. Similarly key G_1_ checkpoint arrest genes, such as CDKN2B, RBL2, CDK5RAP1, and CCNC, coincided with the delayed G_1_/S transition observed in CP CML progenitors, which also harbored longer G_1_ transit times. Significant downregulation of both ATM and RAD9A, two key DNA damage response genes, suggests that CP CML progenitors in G_1_ phase are more susceptible to DNA damage. Analysis of the S/G_2_/M cell cycle phases revealed 17 differentially expressed genes (Fig. 3f). Of these genes, MCM2 and CCNF were significantly (p < 0.05) upregulated in CP CML versus normal progenitor cells suggesting that two key processes that distinguished CP CML from normal progenitors were regulation of DNA replication and repair.

## Discussion

In contrast to dormant hematopoietic stem cells, which tightly regulate DNA damage responses^21^,^22^,^23^,^24^, slowly cycling malignant progenitors in CML persist despite accumulation of DNA double strand breaks and unfaithful repair^25^,^26^,^27^,^28^,^29^. In keeping with these findings, our RNA-seq analysis revealed that KEGG cell cycle, DNA replication and DNA mismatch repair pathways were significantly differentially regulated between CP CML and normal progenitors. Together these studies provided a compelling rationale for developing reliable and robust methods to investigate niche-dependent and cell autonomous drivers of cell cycle transit and cell cycle regulatory gene expression in live normal and malignant progenitors.

Thus, we developed a lentiviral bicistronic fluorescent ubiquitination-based cell cycle indicator (Fucci2BL) system that enables efficient one-step transduction of normal progenitors and patient-derived malignant progenitors as well as real-time quantification of single live progenitor cell cycle kinetics in response to a defined niche. The Fucci2BL lentiviral reporter system facilitates fluorescent quantification of G_1_, G_1_/S, and S/G_2_/M cell cycle phases and real-time clonal tracking by confocal fluorescence microscopy analyses. The reporter also facilitated the purification of live cells in different cell cycle stages, potentially allowing for the enrichment of slow cycling cells, that may be resistant to therapies that target rapidly dividing cells and thus promote relapse.

Development of the single transduction Fucci2BL system enabled FACS-purification and gene expression profiling of human normal and CP CML progenitors in the G_1_ phase of the cell cycle after co-culture on a defined niche. In contrast to their normal progenitor counterparts, G_1_ CP CML progenitors showed decreased expression of DNA damage response genes required for accurate double strand DNA break repair, such as ATM and RAD9A^30^. Coupled with previous findings that BCR-ABL promotes genomic instability through DNA damage and inefficient recombination repair, our results suggest that decreased expression of DNA damage response genes in G_1_ phases may contribute to malignant progression and resistance^29^,^31^,^32^.

Distinctive niche-responsive alterations in gene expression between normal and CP CML progenitors were also detected in S/G_2_/M cell cycle phases. In particular, MCM2, a licensing factor that regulates DNA replication in S phase, was found to be significantly upregulated in CP CML compared with normal progenitors. Moreover, expression of an F box protein CCNF, which mediates genome stability through regulation of dNTP levels,^33^ was also increased in S/G_2_/M phase of the cell cycle in CP CML compared with normal progenitors. Cumulatively, these data suggest that malignant progenitors have an enhanced capacity for DNA replication despite DNA damage acquired as a consequence of DNA repair defects in the G_1_ phase of the cell cycle. Detection and characterization of niche-responsive and cell autonomous regulators of cell cycle transit in live normal and malignant progenitors may inform development of targeted CSC eradication strategies in a broad array of malignancies and thus may help to obviate relapse.

As expected in confocal fluorescence microscopic imaging studies of live cells on a defined niche, cells migrate off the niche depending on cell cycle status. However, cells can be further analyzed by FACS and PCR array to determine cell cycle status and cell cycle-dependent gene expression characteristics, respectively. Notably, our novel Fucci2BL single cell cycle tracking system captures an important aspect of leukemia stem cell biology in that CP CML progenitors are more likely to adhere to the niche and take longer to transit the G_0_/G_1_ phase of the cell cycle compared with their normal progenitor counterparts. Finally, the Fucci2BL system will enable accurate real-time evaluation of small molecule inhibitors of primary human leukemia stem cell dormancy in the niche that promote sensitization to therapeutic agents targeting dividing cells.

## Methods

### Whole transcriptome RNA sequencing

RNA sequencing was performed using purified RNA from 50,000 CD34^+^ selected progenitor cells from each sample type normal cord blood (n = 3), normal peripheral blood (n = 3), and chronic phase CML (n = 8) as described previously^2^,^34^.

### Gene Set Enrichment Analysis

A Gene Set Enrichment Analysis (GSEA) using Kyoto Encyclopedia of Genes and Genomes (KEGG) (http://www.genome.jp/kegg/kegg1.html) gene sets was performed on 30,325 gene FPKM values per sample for 3 NP, 3 CB, and 8 CP CML samples, comparing CP CML samples versus the other samples as described previously^1^. A heatmap was produced from the top 75 genes by absolute GSEA feature score using GSEA software version 2.2.0 (http://www.broadinstitute.org/gsea).

### Ingenuity Pathway Network Analysis

Log2-fold change was calculated for CP CML (n = 8) versus normal control (n = 6, 3 NP, 3 CB) samples based on RNA-seq gene expression values in FPKM, then imported to Ingenuity Pathway Analysis (IPA) for network assembly. For both networks genes upregulated in CP CML are colored red, while genes downregulated in CP CML are colored green.

### Cell Culture

293A cells were cultured in DMEM (Corning Cell Grow, cat. 10–017-CV) supplemented with 10% (v/v) FBS (Gemini, cat. 100—106) and 1% (v/v) penicillin-streptomycin. Both M2 and SL cells were purchased (StemCell Technologies). M2 cells were cultured in RPMI supplemented with 10% (v/v) FBS and produce both human interleukin-3 (IL-3) and human granulocyte colony stimulating factor (G-CSF). SL cells produce (IL-3) and human stem cell factor (SCF). SL cells were cultured in DMEM supplemented with 10% (v/v) FBS and selected every three passages with 0.8 mg/ml G418 (Life Technologies, cat. 11811031), 0.125 mg/ml Hygromycin B (StemCell Technologies, cat. 03813). M2 cells were cultured in RPMI (GIBCO, cat 11875 93) supplemented with 10% (v/v) FBS and selected every three passages with 0.4 mg/ml G418, 0.06 mg/ml Hygromycin B. Primary patient CD34^+^ selected chronic phase CML and normal peripheral blood samples were cultured in Myelocult H5100 (StemCell Technologies, cat. 05150) supplemented with the following cytokines and ligands from R&D systems, 50 ng/mL stem cell factor (SCF, cat. 255-sc), 10 ng/mL Thrombopoietin (TPO, cat. 288-TP), 50 ng/mL fms-like tyrosine kinase-3 (FLT3, cat. 308-FK), and 10 ng/mL interleukin-6 (IL-6, cat. 206-IL) as previously described^2^,^3^,^34^,^35^.

### Stromal Co-Culture System for Real-time Live Progenitor Cell Cycle Imaging

Confluent SL and M2 cells were harvested with 0.05% Trypsin (Life Technologies), and then irradiated with 8000 cGy of ionizing radiation. Following irradiation cells were incubated for 2 hours then counted with trypan blue and plated at a ratio of 1:1 at 2.5 × 10^5 ^cells/ml into Mattek 35 mm with 14 mm Microwell No.1.5 glass bottom, poly-d-lysine coated dishes (Part # P35GC-1.5-14-C). On day two CD34^+^ selected normal progenitors (NP) or chronic phase CML progenitors (CP) cells were plated on top of the adherent SL/M2 cells and cultured in Myelocult H5100.

### Plasmid Construction

The bicistronic lentiviral Fucci2BL expression vector was generated by subcloning both mVenus-hGem(1/110) and mCherry-hCdt1(30/120) into pCDH-T2A-copGFP (CD521A-1, SBI Systems Biosciences). The first step involved ligating the PCR product mCherry-hCdt1(30/120) into BspE1/SalI digested pCDH-EF1-T2A-copGFP. Ligating mCherry-hCdt1(30/120) into BspEI/SalI digested pCDH-T2A-copGFP in-frame replaced copGFP with mCherry-hCdt1(30/120). The second step involved ligating the PCR product mVenus-hGem(1/110) into XbaI/BamHI digested pCDH-EF1-T2A- mCherry-hCdt1(30/120). Ligating mVenus-hGem(1/110) into XbaI/BamHI digested pCDH-T2A- mCherry-hCdt1(30/120) linked mVenus-hGem(1/110) and mCherry-hCdt1(30/120) by 2A peptide. Both pCSII-EF- mVenus-hGem(1/110) and pCSII-EF-mCherry-hCdt1(30/120) were used as template for both rounds of PCR. The first round of PCR used forward primer: BspE1/ mCherryhCdt1 (5′-GGA CCT TCC GGA ATG GTG AGC AAG GGC-3′) and reverse primer SalI/mCherryhCdt1 (5′-ACG CGT CGA CTT AGA TGG TGT C-3′). The second round used forward primer: XbaI/mVenus-hGeminin (5′-TGC TCT AGA GCC ACC ATG GTG AGC AAG GGC and reverse primer: BamHI/hVenusGeminin (5′-CGG GAT CCC AGC GCC TTT CTC-3′). The housekeeping elongation factor 1α (EF1) promoter drives the expression of both mVenus-hGem(1/110) and mCherry-hCdt1(30/120) in the bicistronic Fucci2BL lentiviral reporter vector. Primers used in this study synthesized by ValueGene (San Diego, CA) are summarized in (Supplementary Fig. 2c). Restriction enzyme analysis (Supplementary Fig. 2d) and DNA sequencing were used to verify Fucci2BL plasmid.

### CD34^+^ Selection of Primary Patient Sample

Untreated chronic phase (CP) CML samples were obtained from patient peripheral blood that was collected with informed consent for research use in accordance with Institutional Review Board approved protocols at UCSD or purchased from AllCells (Alameda, CA). Normal peripheral blood mononuclear cells (PBMCs) were purchased from the San Diego Blood Bank. Peripheral blood mononuclear cells (PBMCs) were isolated by Ficoll (GE Healthcare, cat# 17-1440-03) density centrifugation and viable cells were stored in liquid nitrogen in freezing media. CD34^+^ cells were enriched from both (CP) CML and NP by immunomagnetic bead separation following manufacturer’s instructions (MAC; Miltenyi, Bergisch Gladbach, Germany) as described previously^1^,^2^,^3^,^35^. The selected cells were collected and resuspended in 1ml of MyeloCult supplemented with cytokines and cultured for 24 hours before they were counted and transduced.

### Fucci2BL Transduction of CD34^+^ Progenitor Cells

Transduction of both normal and CML CD34^+^ enriched progenitors was performed as follows: 50,000 cells/100uL were plated in 96-well U-bottom plates in MyeloCult (Life Technologies) supplemented with human cytokines (IL-6 10 ng/mL, FLT3 50 ng/mL, SCF 50 ng/mL, and TPO 10 ng/mL). Cells were transduced with a multiplicity of infection (MOI) of 200 Fucci2BL for 72 hours.

### Cell Cycle FACS Analysis

For cell cycle FACS analysis, 293A cells were harvested with Accutase (StemCell Technologies), and washed with 10ml PBS with 1%FBS. The cell pellet was washed with 10ml PBS and fixed with 100 μL 1% (v/v) PFA in PBS for 15 min at 4 °C. Then, 2 mL of 70% ethanol were added dropwise while vortexing and cells were incubated for 2 hours at −20 °C. Cells were then washed twice with 2 ml PBS with 1%FBS supplemented with 0.2% saponin. Alexa Fluor Ki67-A647 (BD Pharmingen cat# 561126 Clone B56) was diluted 1:50 with PBS with 1%FBS supplemented with 0.2% saponin and 50 μL were added to the cells, and incubated for 45 min at room temperature. Cells were washed with 2ml PBS with 1%FBS with 0.2% saponin and stained with DAPI, which was diluted 1:10,000 and incubated for 10 min at room temperature. Cells were washed with PBS with 1%FBS and analyzed on a FACS Fortessa analyzer.

### Live Cell Staining

While 293A cells were grown in a 10 cm plate, 56 μL of 1 mg/mL Hoechst were added to 10 mL of DMEM with 10% FBS and cells were incubated in a 37 °C incubator with 5% CO2 for 30 min. Harvested cells were analyzed using a FACS Fortessa. 293A cells transfected with either mCherry-hCdt1(30/120) (ex 587 em 610) or mVenus-hGem(1/110) (ex 396/475 em 508) were used as controls.

### Time lapse confocal fluorescence microscopic imaging

Confocal fluorescence images were acquired using the Olympus Confocal Laser Scanning Microscope Model FLUOVIEW FV10i-DOC equipped with UPLSAP10X/0.4-NA objective and stage top incubator (cat# INUG2-FV10i, TOKAI HIT, Japan). The Digital Gas Mixer maintained a humidified atmosphere at 37 °C, with 5% CO_2_. Images were taken with an image size 1024 × 1024(pixel), Balance (×4) speed & quality (average), ×2.5 confocal aperture with crosstalk correction on. The excitation spectral values were as follows: ex 473 nm (power at 4.9%, green channel) and ex 559 nm (power at 4.9%, red channel). Emission spectral ranges were as follows: Green-Narrow Em490–540 and Red-Narrow Em570-620. Time-lapse imaging was acquired taking phase contrast, red and green fluorescent recorded images every 15 minutes for over 30 hours of imaging for each NP and CP CML co-cultures. After completion of time-lapse imaging an animation was complied with all images acquired every 15 minutes. Cell cycle kinetics of both NP and CP CML cells was determined using Olympus Fluoview Ver.2.1c analysis software. Single cells were tracked independently throughout animation and single measurements were made from images taken every 15 minutes. Specifically, measurements were made using analysis tools by defining a region of interest (ROI) using the tool view window on each individual cell. The average red and green intensity was recorded from every image throughout the entire time-lapse animation. Cell cycle kinetics were determined by plotting average red and green fluorescent intensity over time using Prism software. The Fluoview FV10i raw images were reconstructed using ImageJ Software.

### Cell Cycle PCR Array

Freshly sorted cells were collected directly into RLT lysis buffer (Qiagen, Germantown, MD). RNA was purified using RNeasy extraction kit with an on column DNase digest step to remove trace genomic DNA present. RNA concentrations were determined using a NanoDrop 2000 spectrophotometer (Thermo Scientific). RNA was stored in −80 °C until all samples were sorted and purified. Equal amounts of RNA (16ng/sample) were converted into cDNA using the First Strand cDNA Synthesis (Qiagen) kit. Due to individual sample RNA concentrations of 1–13 ng/μL, a Preamplification of cDNA Target Templates for the RT2 Profiler PCR Cell Cycle Array (Qiagen) was completed. The PCR Array profiled the expression of 84 cell cycle genes, 5 housekeeping genes, controls for genomic DNA contamination, and efficiency of both the (RT-PCR: PCR) reactions. Cycling conditions for amplification of cDNA from fresh/frozen samples were followed according to manufactures protocol. Cell cycle gene expression was determined using the BioRad CFX96 system (UCSD Human Stem Cell Core Facility).

### Statistical analysis

Statistical analyses were performed with Microsoft Excel, R, Graphpad Prism software, and SAS statistical software.

## Additional Information

**How to cite this article**: Pineda, G. *et al*. Tracking of Normal and Malignant Progenitor Cell Cycle Transit in a Defined Niche. *Sci. Rep*. **6**, 23885; doi: 10.1038/srep23885 (2016).

## Supplementary Material

Supplementary Information

Supplementary Movie S1

Supplementary Movie S2

Supplementary Movie S3
