# Supplementary material for: Tracking of Normal and Malignant Progenitor Cell Cycle Transit in a Defined Niche
Source: Sci Rep. 2016 Apr 4;6:23885. doi: 10.1038/srep23885 (PMC4819192; doi:10.1038/srep23885)
Supplement: Supplementary Information [file srep23885-s1.pdf]

Scientific Reports

Supplementary Figures

Tracking of Normal and Malignant Progenitor Cell Cycle Transit in a Defined Niche

Gabriel Pineda[1], Kathleen M. Lennon[1], Nathaniel P. Delos-Santos[1], Florence Lambert-Fliszar[1], Gennarina L. Riso[1,2], Elisa Lazzari[1,3], Marco A Marra[4], Sheldon Morris[1], Asako Sakaue-Sawano[5], Atsushi Miyawaki[5], and Catriona H. M. Jamieson[1, a]

a.

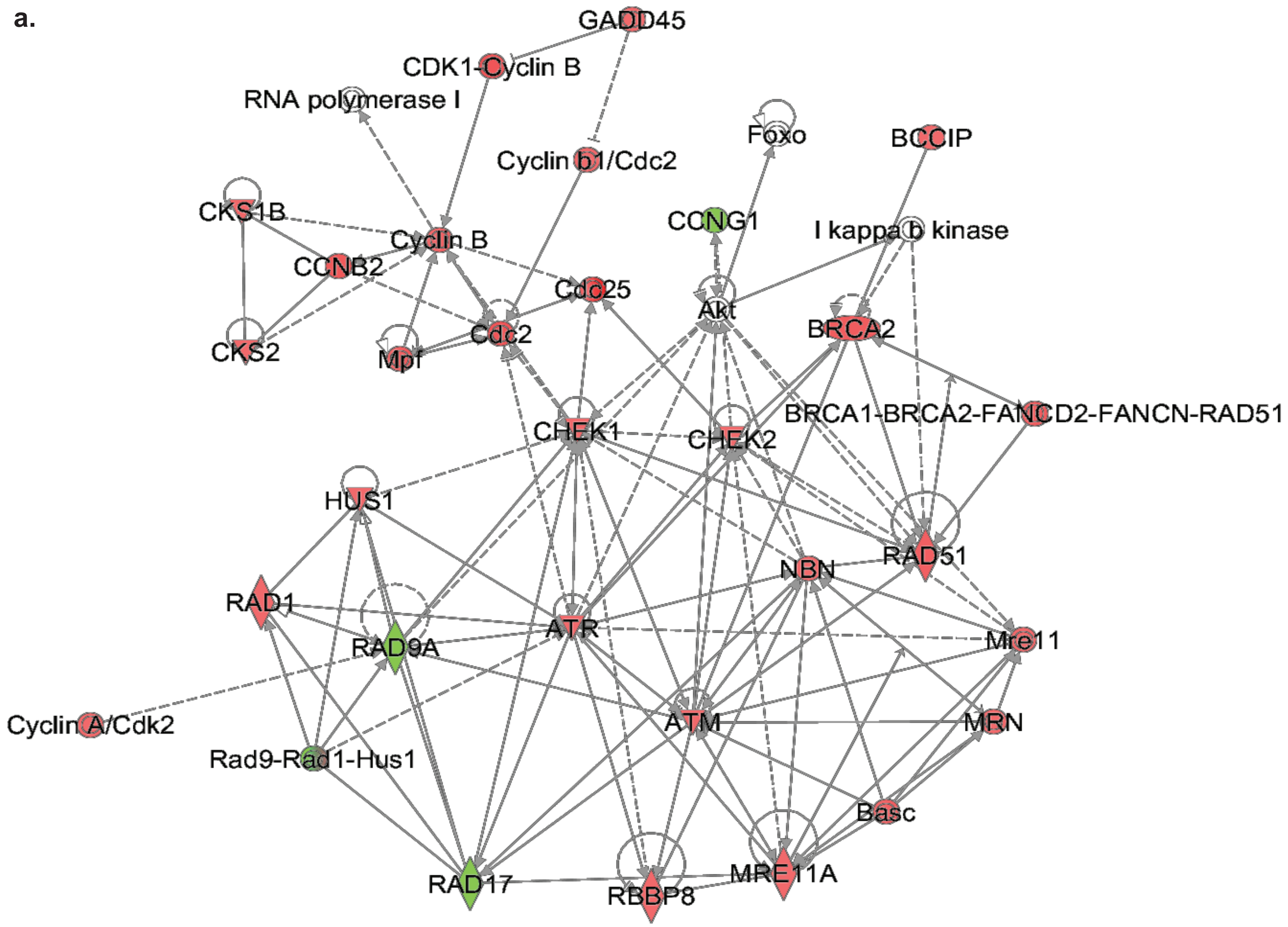
GADD45
CDK1-Cyclin B
RNA polymerase I
Cyclin b1/Cdc2
Foxo
BCCIP
CKS1B
CCNG1
I kappa b kinase
Cyclin B
CCNB2
Cdc25
Akt
BRCA2
CKS2
Mpf
Cdc2
BRCA1-BRCA2-FANCD2-FANCN-RAD51
CHEK1
CHEK2
HUS1
RAD51
NBN
RAD1
ATR
RAD9A
Mre11
Cyclin A/Cdk2
ATM
MRN
Rad9-Rad1-Hus1
Basc
MRE11A
RAD17
RBBP8

a.

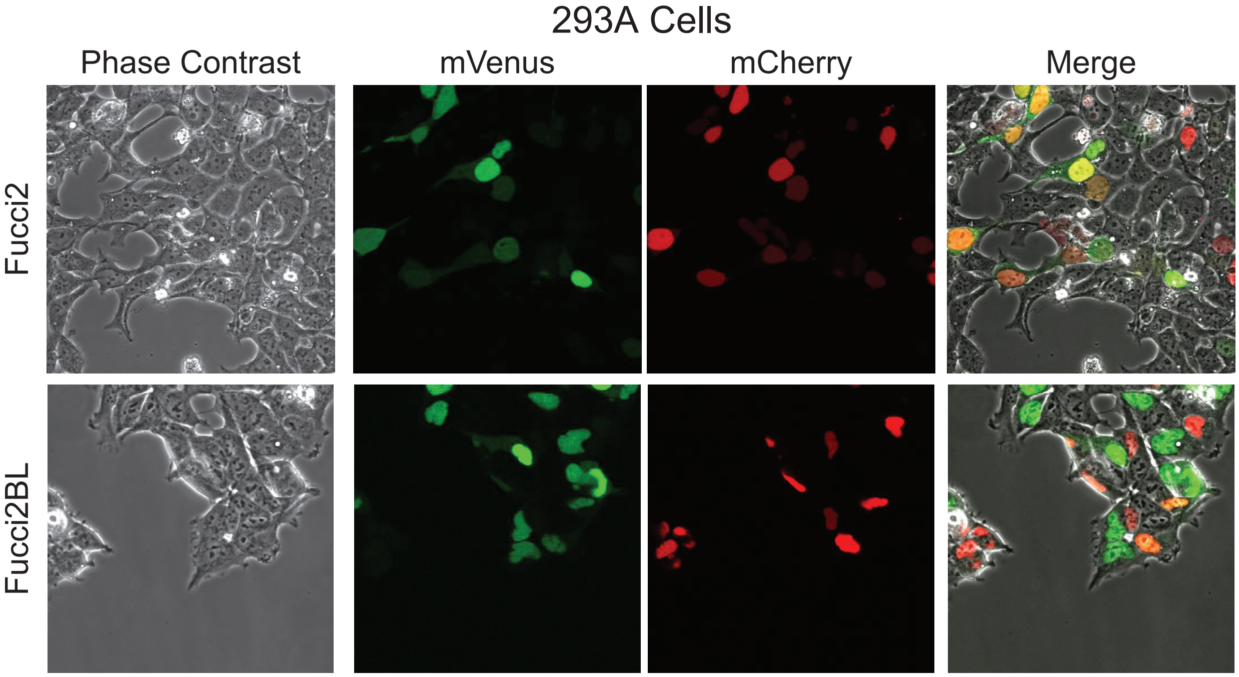

b.

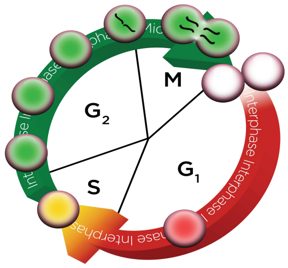

c.

Primers	
5'BspE1 / mCherryhCdt1	GGACCTTCCGGAATGGTGAGCAAGGGC
3'SalI / mCherryhCdt1	ACGCGTCGACTTAGATGGTGTC
5'XbaI / mVenushGem	TGCTCTAGAGCCACCATGGTGAGCAAGGGC
3'BamHI / mVenushGem	CGGGATCCCAGCGCCTTTCTC

d.

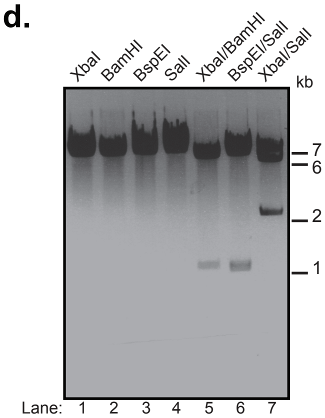

e.

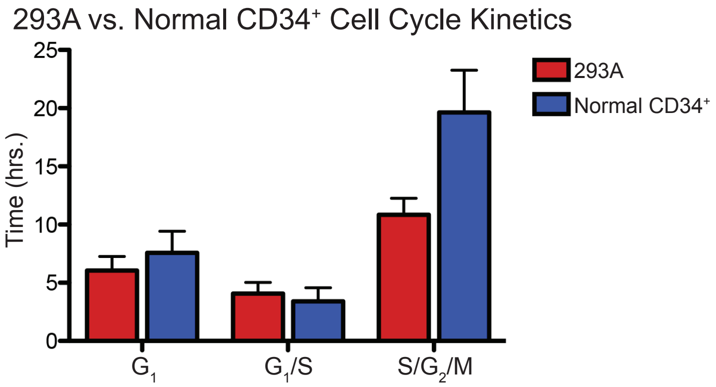

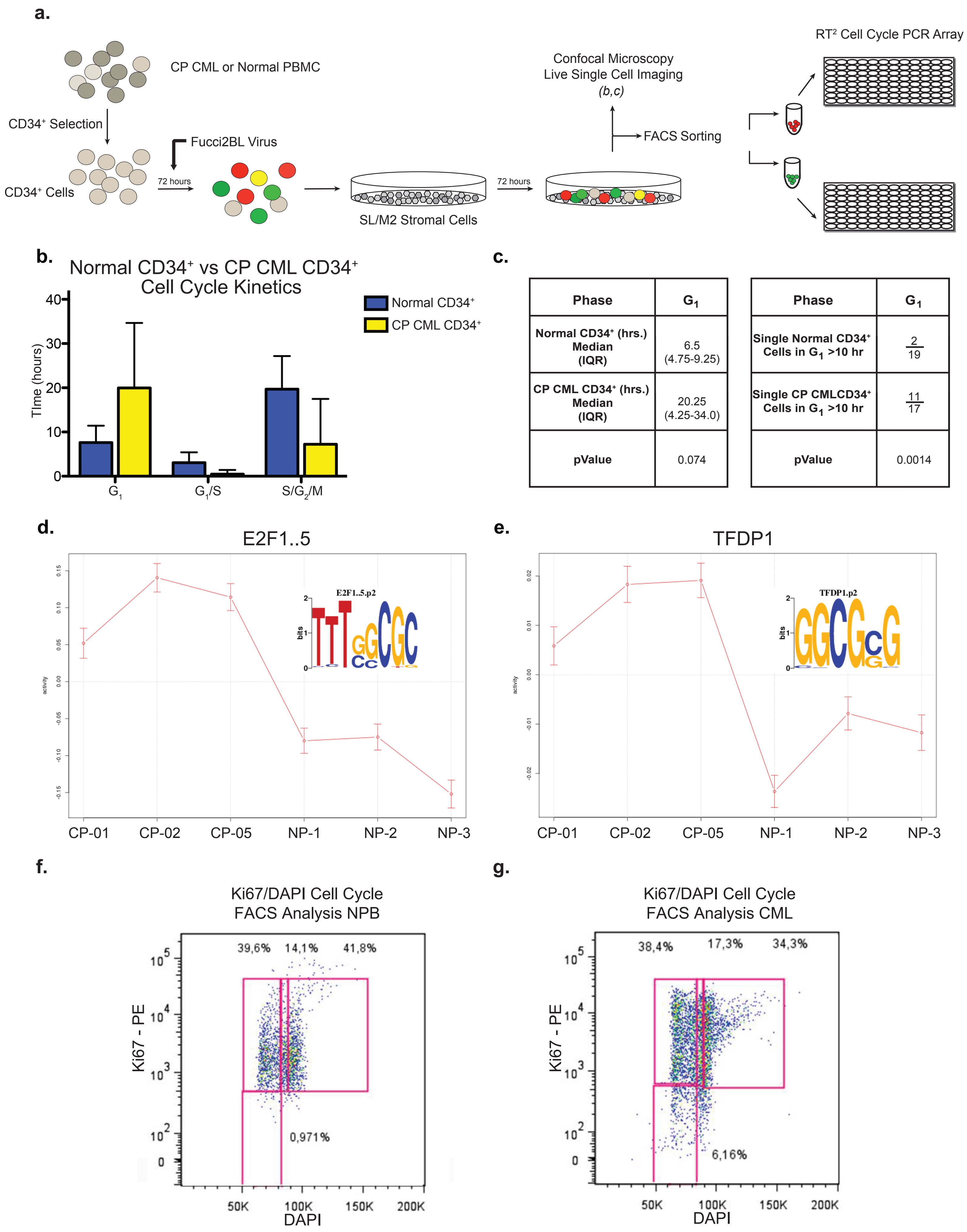
a.
CP CML or Normal PBMC
CD34+ Selection
CD34+ Cells
72 hours
Fucci2BL Virus
SL/M2 Stromal Cells
72 hours
Confocal Microscopy
Live Single Cell Imaging
(b,c)
FACS Sorting
RT2 Cell Cycle PCR Array
b.
Normal CD34+ vs CP CML CD34+
Cell Cycle Kinetics
Normal CD34+
CP CML CD34+
TIme (hours)
40
30
20
10
0
G1
G1/S
S/G2/M
c.
Phase
G1
Normal CD34+ (hrs.) Median (IQR)
6.5 (4.75-9.25)
CP CML CD34+ (hrs.) Median (IQR)
20.25 (4.25-34.0)
pValue
0.074
Phase
G1
Single Normal CD34+ Cells in G1 >10 hr
2/19
Single CP CMLCD34+ Cells in G1 >10 hr
11/17
pValue
0.0014
d.
E2F1..5
E2F1..5.p2
bits
activity
CP-01
CP-02
CP-05
NP-1
NP-2
NP-3
e.
TFDP1
TFDP1.p2
bits
activity
CP-01
CP-02
CP-05
NP-1
NP-2
NP-3
f.
Ki67/DAPI Cell Cycle
FACS Analysis NPB
39,6%
14,1%
41,8%
0,971%
Ki67 - PE
DAPI
50K
100K
150K
200K
g.
Ki67/DAPI Cell Cycle
FACS Analysis CML
38,4%
17,3%
34,3%
6,16%
Ki67 - PE
DAPI
50K
100K
150K
200K

Supplementary Figure 1. Ingenuity®Pathway analysis of CP CML compared to NP cells from Cell Cycle PCR array genes.

Ingenuity®Pathway analysis of CP CML compared to NP from Cell Cycle PCR array gene sets. Red indicates increased expression and green indicates decreased expression in CP relative to NP.

Supplementary Figure 2. Characterization of Fucci2BL Reporter.

(a) Comparison of cell cycle reporters in 293A cells co-transduced with each independent Fucci2 reporter (mVenus-hGem(1/110) and mCherry-hCdt1(30/120)) or single transduction with Fucci2BL lentivirus reporter. (b) Cartoon depicting colors generated by the fluorescent reporters in each phase of the cell cycle. (c) DNA primer sequences used to subclone both hGem(1/110) and hCdt1(30/120) into lentiviral expression vector pCDH EF1α T2A. (d) Sequence validation of, bicistronic lentiviral reporter vector by restriction enzyme digest. (e) Comparision of cell cycle kinetics between 293A cells and normal peripheral blood $CD34^+$ progenitor cells.

Supplementary Figure 3. Cell Cycle Array Identifies Distinct Differences in Normal and Chronic Phase CML Progenitor cell cycle regulatory gene expression.

(a) Experimental strategy used to isolate (G_1) or ($S/G_2/M$), cells for gene expression analysis and live cell imaging using confocal microscopy. (b) Comparison of cell cycle kinetics between normal peripheral blood and CP CML $CD34^+$ progenitor cells. (c) Comparison and analysis of the G_1 phase of kinetics between NP (n=19) and CP CML (n=17) cells represented by median (hours). Interquartile range (IQR) between NP (n=19) cells vs. CP CML (n=17) G_1 phase and significance represented by p-value using average time of cell cycle phases represented in hours. (d) E2F1-5 transcription factor binding motif with the highest activity on gene expression changes between CP and NP using ISMARA. (e) E2F1-5 transcription factor binding motif with the second highest activity on gene expression changes between CP and NP using ISMARA. (f) Cell cycle FACS analysis using Ki-67 staining and DAPI on normal peripheral blood $CD34^+$ cells. (g) Cell cycle FACS analysis using Ki-67 staining and DAPI on CP CML $CD34^+$ cells.

Supplementary Video 1. Characterization of 293A cell cycle kinetics using the Fucci2BL reporter. Time-lapse imaging using confocal fluorescence microscopy of 293A cells transduced with the Fucci2BL reporter, which stably express mVenus-hGem(1/110) and mCherry-hCdt1(30/120).

Supplementary Video 2. Characterization of NP cell cycle kinetics using the Fucci2BL reporter. Time-lapse imaging using confocal fluorescence microscopy of $CD34^+$ selected normal human progenitors (NP) transduced with

the Fucci2BL reporter followed by culturing on a SL/M2 stromal co-culture system.

Supplementary Video 3. Characterization of CP cell cycle kinetics using the Fucci2BL reporter. Time-lapse imaging using confocal fluorescence microscopy of $CD34^+$ selected chronic phase CML human progenitors (CP) transduced with the Fucci2BL reporter followed by culturing on a SL/M2 stromal co-culture system.
